# Association of low back load with low back pain during static standing

**DOI:** 10.1371/journal.pone.0208877

**Published:** 2018-12-18

**Authors:** Tetsuya Hasegawa, Junji Katsuhira, Hiroyuki Oka, Tomoko Fujii, Ko Matsudaira

**Affiliations:** 1 Rehabilitation Center, Tochigi Medical Association Shiobara Spa Hospital, Nasushiobara-shi, Tochigi, Japan; 2 Faculty of Medical Technology, Department of Prosthetics and Orthotics and Assistive Technology, Niigata University of Health and Welfare, Kita-ku, Niigata-shi, Niigata, Japan; 3 Department of Medical Research and Management for Musculoskeletal Pain, 22nd Century Medical and Research Center, Faculty of Medicine, The University of Tokyo, Bunkyo-ku, Tokyo, Japan; University of Münster, GERMANY

## Abstract

**Background:**

Although poor standing posture is a known cause of low back pain, the mechanisms involved are unclear. The aim of this study was to clarify the kinetic and posture angle features of standing posture that might influence low back pain.

**Methods:**

Sixty-seven young men were enrolled in this cross-sectional case-control study and were categorized according to whether they did or did not have low back pain. Habitual standing posture was assessed in each group, using a three-dimensional motion analysis system, force plates, and a spinal mouse. Kinetic and posture angle factors were compared between participants with and without low back pain. The relationship between specific features of standing posture and low back pain was analyzed using logistic regression.

**Results:**

The intervertebral disc compressive force and the low back moment were significantly greater in the group with low back pain than in the group without low back pain. The intervertebral disc compressive force was the factor most strongly associated with low back pain during static standing.

**Conclusions:**

Logistic regression analysis identified intervertebral disc compressive force as an independent variable associated with low back pain. This finding suggests that increased intervertebral disc compressive force may promote development of low back pain in standing posture.

## Introduction

Low back pain is one of the most common health complaints in Japan, Europe, and the United States and is the most frequently occurring occupational health problem. As a major cause of work absenteeism, low back pain incurs substantial economic and social costs [1–3]. A complicated array of anatomical factors, in addition to psychosocial factors and aspects of the workplace environment, are reported to trigger low back pain [4,5]. Habitual poor standing posture may be a risk factor for low back pain in the workplace [6]. Studies of the relationship between back pain and standing posture in the sagittal plane have indicated that posture deviating from the neutral position increases the risk of developing low back pain [7–9].

Poor posture can exert a large mechanical load on the low back. Previous studies have reported that the intervertebral disc compressive force increases with trunk flexion [10–12]. Furthermore, uneven pressure on the intervertebral discs has been reported when the lumbar vertebrae are in a flexed or an excessively extended position [13]. Thus, poor posture may lead to low back pain from a mechanical point of view.

Previous studies on the relationship between standing posture and low back pain have examined pelvic asymmetry [14] and postural changes [15] in standing position but the influence of low back load on the development of low back pain during standing remains unclear.

The aim of this study was to identify the kinetic and posture angle features of standing posture that determine the presence or absence of low back pain.

## Materials and methods

### Study design and setting

This cross-sectional case-control study was conducted between August 2014 and August 2015. The study protocol was approved by the institutional review board of the International University of Health and Welfare. All participants provided written informed consent.

### Participants

The participants were 67 male university students with a mean age of 23.9 ± 3.3 years, a mean height of 172.7 ± 6.2 cm, and a mean weight of 65.2 ± 7.9 kg.

### Inclusion and exclusion criteria

**Fig 1:**
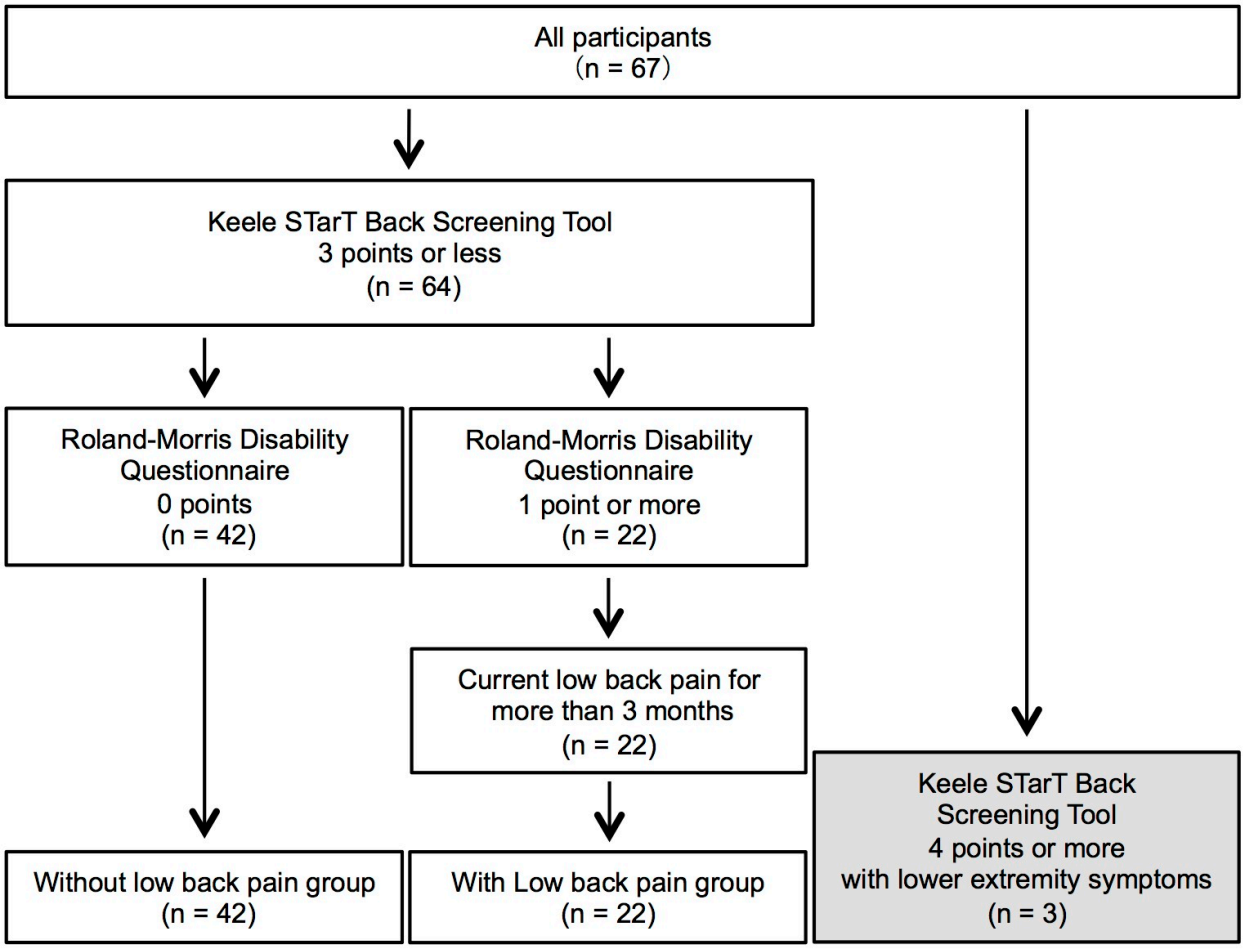
Flow of participants through the trial.

Participants with and without low back pain were identified using a questionnaire on low back pain [16,17], the Roland-Morris Disability Questionnaire (RDQ) [18,19], and the Keele STarT Back Screening Tool (SBST) [20]. Participants with low back pain were defined as those who scored 1 point or higher on the RDQ and had had low back pain for 3 consecutive months or longer. Participants without low back pain were defined as those who scored 0 on the RDQ. Those participants who scored 4 points or more on the SBST were defined as having psychosocial low back pain and were excluded (Fig 1). To exclude low back pain due to lumbar radiculopathy, those with lower limb symptoms were excluded on the basis of their SBST responses. This left 64 participants available for inclusion in the study (Table 1).

**Table 1:** Demographic characteristics of the study population.

	All (n = 64)	Without low back pain (n = 42)	With low back pain (n = 22)
Age, years	23.9 (3.3)	23.9 (3.3)	24.2 (3.1)
Height, cm	172.8 (6.1)	172.4 (6.1)	173.4 (6.3)
Body weight, kg	65.3 (7.9)	64.6 (7.4)	67.2 (8.8)

Data are shown as the mean (standard deviation).

### Experimental setup

**Fig 2:**
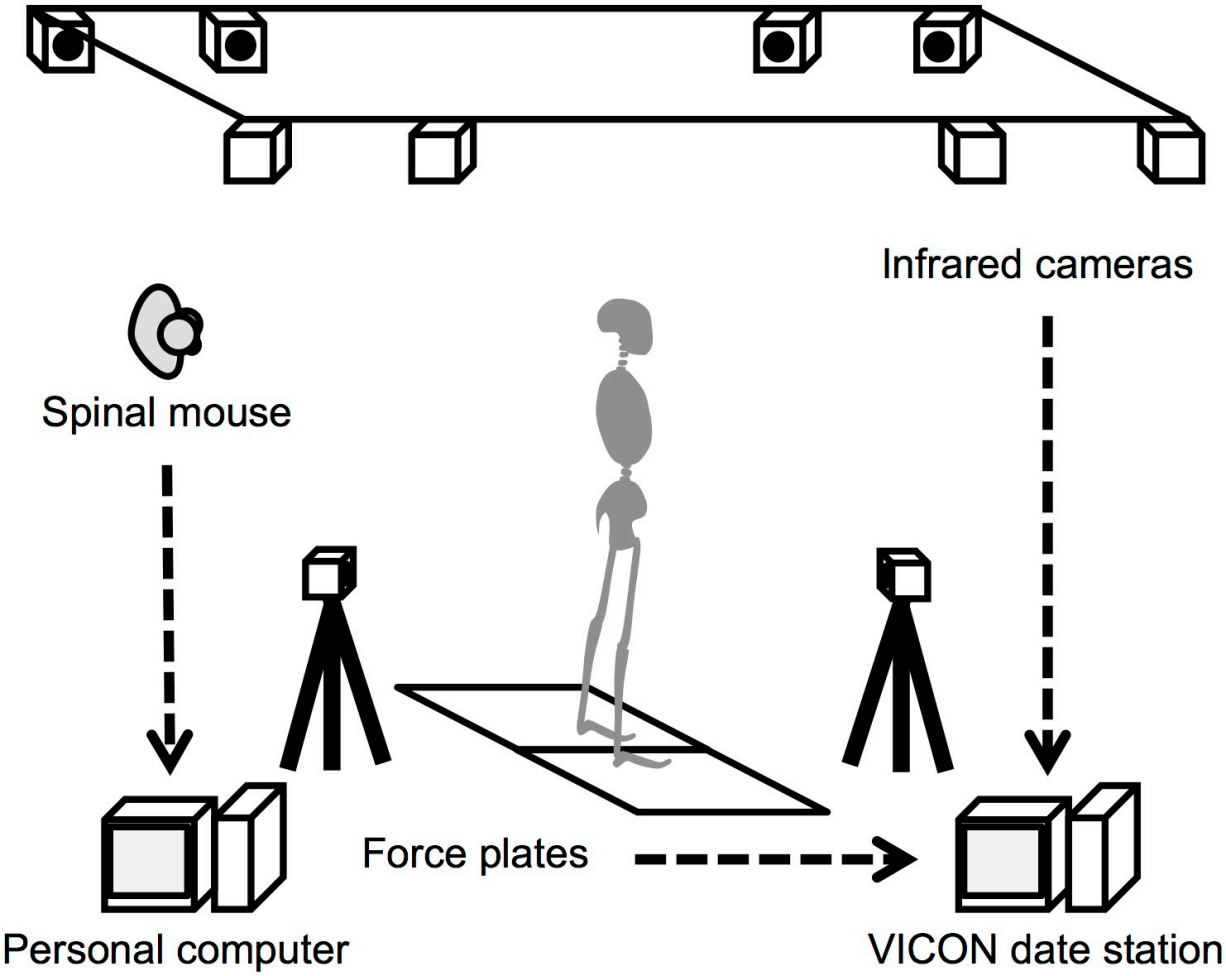
Experimental setup.

Standing posture was measured using a three-dimensional (3D) motion analysis system with 10 infrared cameras (Vicon MX, Vicon Motion Systems Ltd., Oxford, UK), two force plates (AMTI, Watertown, MA), and a spinal mouse (Idiag AG, Fehraltorf, Switzerland). The force plates and the 3D motion analysis apparatus each had a sampling frequency of 100 Hz (Fig 2).

### Experimental conditions

Measurements were taken while standing with the feet placed a shoulder width apart on separate force plates and looking at an eye-level target located 5 m ahead. Habitual standing posture was measured for 10 s using a Vicon motion capture camera. Measurements of standing posture using a spinal mouse were made immediately after the Vicon measurements. The interval between each measurement was 1 min, and repeated stepping motions (i.e., walking in place) were performed between measurements. The measurements were done three times in total.

Infrared reflective markers (diameter 14 mm) were placed on the body in the following locations: top of the head, seventh cervical vertebra, tenth and twelfth thoracic vertebrae, fifth lumbar vertebra, sacrum, manubrium, xiphoid process, side of the head, acromion process, medial epicondyle of the humerus, lateral epicondyle of the humerus, radial styloid process, ulnar styloid process, iliac crest, anterior superior iliac spine, posterior superior iliac spine, hip joint, greater trochanter, medial and lateral sides of the knee, medial malleolus, lateral malleolus, first metatarsophalangeal joint, fifth metatarsophalangeal joint, and calcaneus. All markers were applied by the same examiner.

A spinal mouse was used to measure the spinal curvature angle [21]. This is a small handheld device that measures the curvature of the spine through the motion of two wheels along the spinal column. It was placed along the line of the spinal column, starting at the spinous process of the seventh cervical vertebra and finishing at the third sacral vertebra, the location of which was confirmed by palpation and marked with an infrared reflective marker. Spinal curvature data were transmitted wirelessly to a personal computer and analyzed using dedicated analysis software. All spinal mouse measurements were performed by the same examiner.

### Data analysis

**Fig 3:**
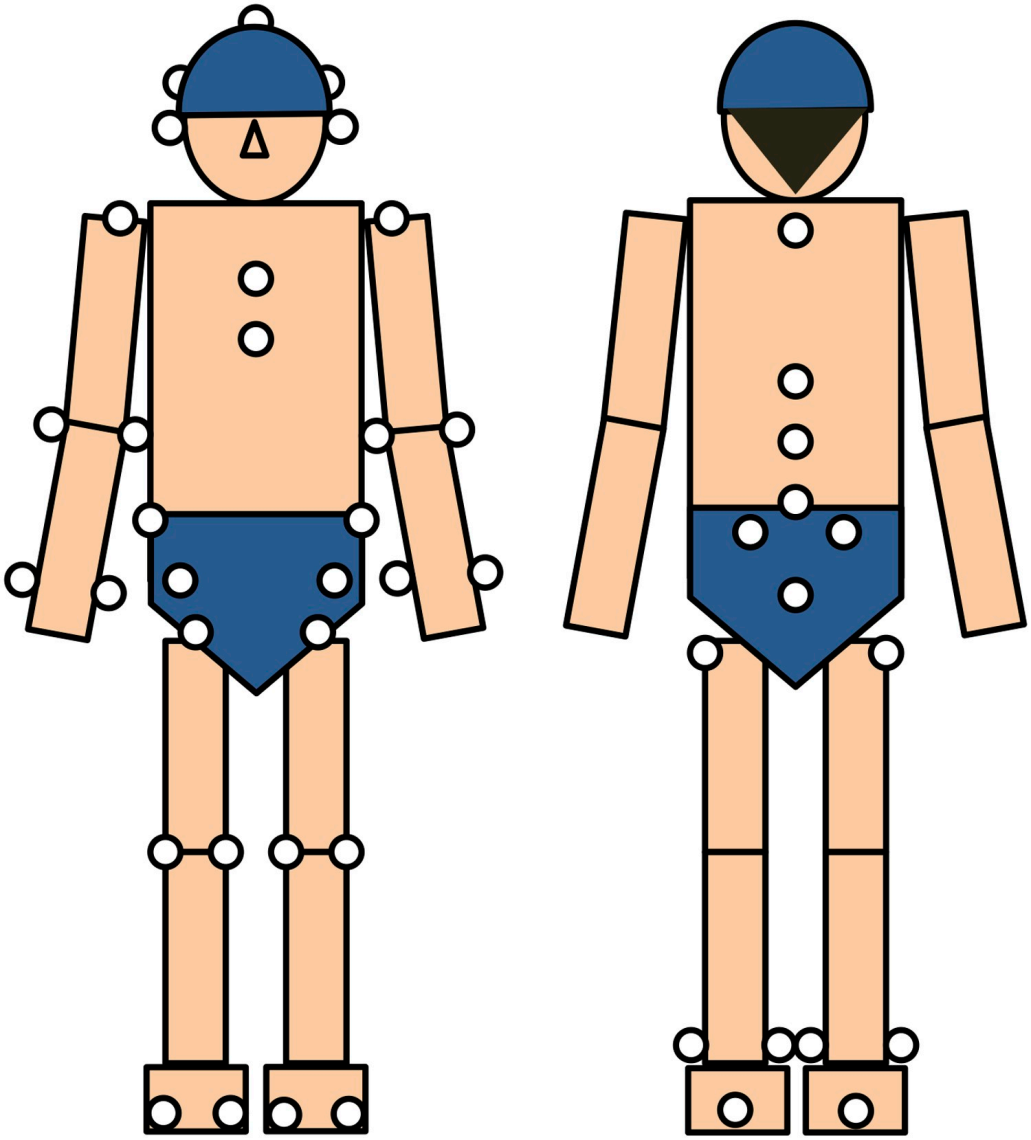
The 13-link segment model and position of infrared reflective markers.

The joint angle and internal joint moment were calculated according to the method described by Katsuhira et al. [22] using the marker positions obtained by the motion capture system and the ground reaction force data obtained from the two force plates. An analysis program was created using the Vicon BodyBuilder 3D motion analysis software (Vicon) and Visual3D version 5 (C-Motion Inc., Germantown, MD). The coordinate and ground reaction force data obtained were low-pass filtered at 6 Hz and 18 Hz, respectively, and used to create a 13-link segment model. The model consisted of head, trunk, and pelvis and bilateral upper arms, forearms, thighs, shanks, and feet (Fig 3). The joint angle was calculated using Euler angles based on the coordinate system defined on each body segment. The joint moment was calculated from the coordinate values of the infrared reflective markers and the ground reaction forces by inverse kinetic analysis using the Newton–Euler method. For the inverse dynamics analysis, the body segments were each regarded as a rigid body and a link segment model was used. In addition to the coordinate position of the joint and the ground reaction force data, the barycentric position, body mass ratio, and moment of inertia of each body segment were required for calculation of the joint moment. Anthropometric parameters for mass, center of mass, and moment of inertia for each segment were obtained from reports by Winter et al. [23], Okada et al. [24], and Jorgensen et al. [25], respectively.

Because low back pain is reported to occur frequently between the 4th and 5th lumbar vertebrae [26], the rotational center of the low back moment was taken between the 4th and 5th lumbar vertebrae in this study.

### Calculation of intervertebral disc compressive force

In addition to the low back moment, the intervertebral disc compressive force between the 4th and 5th lumbar vertebrae was used as an index of the low back load. This force was calculated using the method described by Yamazaki et al. and Hasegawa et al. (Eq 1) [22,27,28]:

$$\begin{array}{l} \text{Intervertebral}\;\text{disc}\;\text{compressive}\;\text{force}\;\\ =20\;|\text{Extension}\;\text{moment}|\;\text{or}\;13\;|\text{Flexion}\;\text{moment}|\\ +8\;|\text{Side}\;\text{flexion}\;\text{moment}|\\ +23\;\left|\text{Rotation}\;\text{moment}\right|\\ +\text{Gravitational}\;\text{force}\;\text{acting}\;\text{on}\;\text{COG}\;\text{of}\;\text{HAT}\;\cdot \text{cos}\theta \end{array}$$ (1)

where *θ* is the trunk angle and HAT means “Head, Arms, Trunk”. The moment arm between the intervertebral disc and the muscle was defined as the distance from the intervertebral disc to the rectus abdominis muscle when the low back flexion moment occurred and as the distance from the disc to the erector spinae muscles when the low back extension moment occurred. The intervertebral disc and the moment arm to each muscle band were described in a previous study [25].

The muscle tension exerted by the paraspinal muscle group was estimated by multiplying the calculated absolute value of the moment applied to the 4th and 5th lumbar vertebrae by the reciprocal of the moment arm, and calculating the gravitational force from the mass of the HAT. After calculation, the forces applied to the 4th and 5th lumbar vertebrae were decomposed in accordance with the trunk angle (*θ*), and the combined forces were taken as the intervertebral disc compressive force (Eq 1).

### Calculation of spinal curvature angle

The spinal mouse and dedicated software (Spinal mouse ver. 3.32, Idiag AG) were used to calculate the spinal curvature angle from the shape of the spinal column and the angle formed by a line perpendicular to the line connecting the upper and lower spinous processes and the adjacent perpendicular. Positive spinal curvature angles indicate kyphosis, whereas negative values indicate lordosis. The curvature of the thoracic vertebrae represents the curvature of the spinal column from the first thoracic vertebra to the 12th thoracic vertebra, that is, the sum of the 11 segmental angles between T1–T2 and T11–T12. The lumbar curve angle represents the curvature of the spinal column from the T12 to the S1, that is, the sum of the 6 segmental angles between T12–L1 and L4–L5 [21].

### Statistical analysis

For the 3D motion analysis and spinal mouse data, the average value of three trials from each participant was analyzed. Joint moment and intervertebral disc compressive force were normalized by body weight. Logistic regression analysis was then used to identify the parameters affecting low back pain. Effect size was calculated using Pearson’s *r*, with effect size values interpreted as 0.10 “small,” 0.30 “medium,” and 0.50 “large” [29]. The presence or absence of low back pain was designated as the dependent variable, and the measured parameters were used as independent variables. Univariate analysis with the unpaired t-test was performed for all variables to select those that predicted low back pain. This was to select independent variables for further analysis, rather than to show significant differences between the groups. Odds ratios and 95% confidence intervals (CIs) were calculated using multivariable (stepwise) logistic regression analysis. Forward selection of variables was performed using the likelihood ratio. Variable selection was conducted using the probability distribution of the likelihood ratio statistic based on maximum biased likelihood estimate. The level of significance was set at 5% for both cases. A Pearson’s product moment correlation coefficient | r | > 0.9 confirmed that there was no correlation between the independent variables. We evaluated the predictive performance of the model by assessing its discrimination (ability to classify correctly). This was measured using the area under the receiver operating characteristic curve (AUC). An area of 1 represents a perfect prediction, while an area of 0.5 represents a completely random prediction. A rough guide for classifying the accuracy of a diagnostic test is the traditional academic point system, with AUC values of 0.90–1 taken as “excellent,” 0.80–0.90 as “good,” 0.70–0.80 as “fair,” 0.60–0.70 as “poor,” and 0.50–0.60 as “fail” [30].

## Results

### Comparison of kinetic and posture angle parameters in participants with and without low back pain

The mean ± standard deviation RDQ score in the group with low back pain was 1.41 0.85. Unpaired *t-*tests revealed no significant differences in height, weight, or posture angle parameters between participants with and without low back pain (Table 2). Among the kinetic parameters, the values for intervertebral disc compressive force and low back moment (flexion/extension) were significantly higher in the group with low back pain than those in the group without low back pain (Table 3).

**Table 2:** Comparison of posture angle parameters between participants with and without low back pain.

			Without low back pain		With Low back pain		*P*-value	Effect size (*r*)
Head angle (deg)	Flexion/extension	Extension+	-12.63	(10.07)	-10.92	(7.27)	0.482	0.10
	Side bending	Right side+	0.74	(2.25)	1.28	(2.27)	0.37	0.12
	Rotation	Right side+	1.51	(3.57)	3.97	(3.71)	0.393	0.32
Spinal curvature (deg)	Thoracic curvature	Kyphosis +	46.99	(6.67)	48.44	(7.72)	0.438	0.10
	Lumbar curvature	Lordosis-	-20.3	(6.44)	-21.65	(8.74)	0.485	0.09
Trunk angle (deg)	Flexion/extension	Extension+	5.16	(3.02)	5.06	(2.91)	0.899	0.02
	Side bending	Right side+	0.04	(1.16)	0.01	(1.73)	0.937	0.01
	Rotation	Right side+	-0.31	(2.57)	-0.26	(2.70)	0.938	0.01
	Pelvic tilt	Anterior-	-7.23	(5.18)	-8.54	(5.30)	0.344	0.12
Right hip angle (deg)	Flexion/extension	Flexion+	-0.44	(6.56)	1.59	(6.45)	0.242	0.15
	Adduction/abduction	Abduction+	1.62	(2.29)	0.69	(2.49)	0.139	0.19
	Rotation	Outward+	-3.6	(6.61)	-0.51	(4.76)	0.056	0.26
Left hip angle (deg)	Flexion/extension	Flexion+	-0.84	(6.66)	1.82	(5.84)	0.119	0.21
	Adduction/abduction	Abduction+	2.02	(2.22)	1.79	(3.39)	0.747	0.04
	Rotation	outward+	-0.4	(5.84)	-2.8	(6.02)	0.128	0.20
Right knee angle (deg)	Adduction/abduction	Flexion+	-2.07	(5.44)	-2.69	(5.82)	0.672	0.05
Left knee angle (deg)	Adduction/abduction	Flexion+	-3.3	(5.38)	-2.61	(5.98)	0.641	0.06
Right ankle angle (deg)	Dorsal/plantar	Dorsal+	1.43	(8.25)	0.41	(1.47)	0.568	0.09
Left ankle angle (deg)	Dorsal/plantar	Dorsal+	1.49	(8.21)	0.56	(1.82)	0.604	0.08

Data are shown as the mean (standard deviation).

**Table 3:** Comparison of kinetic parameters between participants with and without low back pain.

			Without low back pain		With low back pain		*P*-value	Effect size (*r*)
Intervertebral disc compressive force (N/kg)			8.22	(0.79)	9.07	(1.38)	0.012	0.35
Low back moment (Nm/kg)	Flexion/extension	Flexion+	0.18	(0.06)	0.23	(0.08)	0.008	0.33
	Side bending	Right side-	-0.01	(0.05)	0	(0.06)	0.388	0.09
	Rotation	Right side-	-0.01	(0.01)	-0.02	(0.01)	0.514	0.45
Right hip moment (Nm/kg)	Flexion/extension	Extension+	0.11	(0.06)	0.1	(0.06)	0.411	0.08
	Adduction/abduction	Abduction-	-0.05	(0.05)	-0.06	(0.06)	0.223	0.09
	Rotation	Outward+	-0.01	(0.02)	-0.02	(0.02)	0.321	0.24
Left hip moment (Nm/kg)	Flexion/extension	Extension+	0.16	(0.07)	0.16	(0.05)	0.727	0.00
	Adduction/abduction	Abduction-	-0.04	(0.05)	-0.07	(0.07)	0.083	0.24
	Rotation	Outward +	0	(0.01)	-0.01	(0.02)	0.545	0.30
Right knee moment (Nm/kg)	Flexion/extension	Flexion-	-0.09	(0.08)	-0.09	(0.09)	0.894	0.00
Left knee moment (Nm/kg)	Flexion/extension	Flexion-	-0.07	(0.07)	-0.04	(0.08)	0.159	0.20
Right ankle moment (Nm/kg)	Dorsal/plantar	Plantar+	0.26	(0.09)	0.23	(0.08)	0.164	0.17
Left ankle moment (Nm/kg)	Dorsal/plantar	Plantar+	0.22	(0.08)	0.19	(0.07)	0.189	0.20

Data are shown as the mean (standard deviation).

### Logistic regression analysis

Unpaired *t*-tests revealed significant differences between participants with and without low back pain only for the low back flexion and extension moments and intervertebral disc compressive force. Therefore, we performed logistic regression analysis using these parameters as independent variables, with the presence or absence of back pain as the dependent variable. Only intervertebral disc compressive force was selected as a variable (odds ratio = 2.308; Table 4). The chi-square test of the model was significant at *P* < 0.01 (*P* = 0.002).

**Table 4:** Results of logistic regression analysis.

	B	SE	P	OR	95% CI
CF	0.836	0.321	0.009**	2.308	1.229–4.333
Constant	-7.828	2.782	0.005**		

CF: intervertebral disc compressive force. B: unstandardized coefficients. SE: standard error. OR: odds ratio. CI: confidence interval

**: *P* < 0.01

The ROC curve was determined based on the presence or absence of low back pain and the value of the intervertebral disc compressive force, resulting in an AUC of 0.702. The cutoff value was 8.529 (sensitivity 72.7%, false positive rate 33.3%; Table 5). To confirm the superiority of the logistic regression equation using only the intervertebral disc compressive force, we performed logistic regression analysis using only the low back flexion and extension moment and then calculated the AUC. We found that the p-value in the logistic regression was larger than that using only the compressive force and that the AUC of the low back flexion and extension moment dropped below 0.7.

**Table 5:** Cutoff value and AUC.

	Cutoff value	Sensitivity (%)	False positive rate (%)	AUC
CF	8.529	72.7	33.3	0.702

CF: intervertebral disc compressive force

## Discussion

### Comparison of low back load parameters in participants with and without low back pain

In this study, the intervertebral disc compressive force and low back moment about three axes were used as indices of low back load. Nachemson et al. [10], Schultz et al. [11], and Wilke et al. [12] measured this parameter invasively by inserting a pressure gauge directly into the lumbar intervertebral disc while the participant was in a comfortable standing posture.

Nachemson et al. reported values of 0.56 MPa to 0.97 MPa, Schultz et al. reported a value of 0.27 MPa, and Wilke et al. reported a value of 0.50 MPa. These values are equivalent to about 486 N to 1746 N based on a cross-sectional area of 1800 mm^2^ for the vertebral body. The mean intervertebral disc compressive force in the present study was 598.4 ± 114.1 N in the group with low back pain and 524.5 ± 73.8 N in the group without low back pain, which is equivalent to about 0.33 ± 0.06 MPa and 0.29± 0.04 MPa. The value of intervertebral disc compressive force obtained in this study was within the range reported in the previous studies.

Among the low back load indices, the intervertebral disc compressive force and low back moment were significantly larger in the group with low back pain than in the group without low back pain. The weight of the upper body is included in the computation of the compressive force on the intervertebral disc, and thus affects the magnitude of this force. In the present study, however, the value of the intervertebral disc compressive force was normalized by body weight. Moreover, no significant difference in height or weight was found between the groups with and without low back pain. These findings suggest that the increased intervertebral disc compressive force in the group with low back pain was a result of altered posture. However, there were no significant between-group differences in alignment of the trunk and pelvis or in the joint angles and joint moments of the lower limbs, so it was not possible to identify the cause of the increased intervertebral disc compressive force.

### Features of standing associated with the presence or absence of low back pain

Logistic regression analysis was performed using the presence or absence of low back pain as the dependent variable and intervertebral disc compressive force and low back moment (flexion/extension) as the independent variables, which differed significantly between participants with and without low back pain. The results of the logistic regression analysis suggested that the intervertebral disc compressive force contributed to the presence of low back pain.

This analysis revealed that for every 1 N/kg increase in intervertebral disc compressive force, the risk of low back pain increased by about 2.3-fold. In this study, intervertebral disc compressive force was calculated by multiplying the low back moment about three axes by the reciprocals of each moment arm and summing these values to estimate the muscle tension. Using this method, the low back load can be expressed as an increase in the moment about each axis, regardless of the collapse of the posture in either direction.

However, in the logistic regression analysis, the low back moment (flexion/extension) was not identified as a determining factor in the presence or absence of low back pain. This may be because the intervertebral disc compressive force is determined not only by the uniaxial moment but also by the combined effects of the multiple axis moments.

The poor posture of the participants with low back pain was diverse in nature, and the intervertebral disc compressive force was thought to increase because of the complicated association of the moment about each axis, which would affect the likelihood of complaints of low back pain.

The results of this study confirm that individuals with low back pain have higher intervertebral disc compressive force during standing than their counterparts without low back pain. Even though the intervertebral disc compressive force during static standing was small, the increase in this force appeared to contribute to low back pain.

This study has several limitations. First, no women consented to participate in the study, so all the participants were young men. Therefore, it is unclear whether these results are generalizable to other populations, such as women and middle-aged or elderly individuals. To improve the usefulness of this research, the study population should be expanded in future studies. Second, all participants in this study had low average RDQ scores. To further clarify the characteristics of posture in low back pain, it is necessary to accumulate participants with different levels of RDQ scores. Third, because the kinetic parameters measured in this study were obtained via inverse dynamics calculations using a 3D motion analysis device and force plates, the intervertebral disc compressive force is an estimated value. McGill et al. [31] evaluated agonist and antagonist muscle activity using electromyography and calculated intervertebral disc compressive force considering co-contraction of the trunk muscles. However, the equations we used did not take co-contraction into account. In other words, when co-contraction of the trunk muscles occurred, it is possible that we underestimated the compression force of the intervertebral disc. Therefore, it is necessary to validate this method against other low back load estimation methods. Moreover, it may not directly reflect the shape of the intervertebral disc or biological characteristics, such as physiological changes. In addition, although intervertebral disc thickness has been reported to vary diurnally, measurements in this study were taken only during a limited period on a certain day. Fourth, this study did not examine alleviation of low back pain, so we could not determine whether back pain would be improved by a reduction in the intervertebral disc compressive force. This question should be examined by interventional studies in the future.

## Conclusions

This study aimed to identify the kinetic and posture angle features of static standing that affect low back pain. Intervertebral disc compressive force was found to be higher in participants with low back pain, and logistic regression analysis revealed disc compressive force as an independent variable determining the presence or absence of low back pain.

## Supporting information

S1 Table Data of the kinetic and posture angle parameters. (XLSX)
